# Identifying a Novel Defined Pyroptosis-Associated Long Noncoding RNA Signature Contributes to Predicting Prognosis and Tumor Microenvironment of Bladder Cancer

**DOI:** 10.3389/fimmu.2022.803355

**Published:** 2022-01-27

**Authors:** Hongcheng Lu, Jiajin Wu, Linghui Liang, Xinwei Wang, Hongzhou Cai

**Affiliations:** ^1^ Department of Urology, The First Affiliated Hospital of Nanjing Medical University, Nanjing, China; ^2^ Department of Oncology, Jiangsu Cancer Hospital and Jiangsu Institute of Cancer Research and The Affiliated Cancer Hospital of Nanjing Medical University, Nanjing, China; ^3^ Department of Urology, Jiangsu Cancer Hospital and Jiangsu Institute of Cancer Research and The Affiliated Cancer Hospital of Nanjing Medical University, Nanjing, China

**Keywords:** bladder cancer, lncRNAs, pyroptosis, tumor microenvironment, nomogram

## Abstract

**Background:**

Bladder cancer (BLCA) is a common malignant tumor of the urinary tract, which is the sixth most common cancer among men. Numerous studies suggested that pyroptosis and long noncoding RNAs (lncRNAs) played an essential role in the development of cancers. However, the role of pyroptosis-related lncRNAs in BLCA and their prognostic value are still unclear.

**Methods:**

In this study, we constructed a signature model through least absolute shrinkage and selection operator (LASSO) Cox regression analysis and Cox univariate analysis based on The Cancer Genome Atlas (TCGA) database. The expression of 12 pyroptosis-related lncRNAs was also confirmed by qRT-PCR in BLCA cell lines. TIMER, XCELL, QUANTISEQ, MCPCOUNTER, EPIC, and CIBERSORT R script were applied to quantify the relative proportions of infiltrating immune cells. Correlation coefficients were computed by Spearman analyses. The Kaplan–Meier method, Cox regression model, and log-rank tests were used to evaluate the prognostic value. The R package of pRRophetic was used to predict IC50 of common chemotherapeutic agents.

**Results:**

A total of 12 pyroptosis-related lncRNAs with great prognosis value were identified. The expression was investigated by qRT-PCR in four BLCA cell lines. Then, 126 cases were identified as high-risk group, and 277 cases were identified as low-risk group based on the cutoff point. Patients in the low-risk group showed a significant survival advantage. Furthermore, we found that clinical features were significantly related to the risk score. As well, based on the C-index values, a nomogram was constructed. The gene set enrichment analysis (GSEA) results showed that mitogen-activated protein kinase (MAPK) signaling, transforming growth factor (TGF)-β signaling, and WNT signaling were with important significance in the high-risk group. Moreover, we found that riskscore was positively correlated with M0 macrophages and M2 macrophages.

**Conclusions:**

In conclusion, our study indicated that pyroptosis is closely connected to BLCA. The riskscore generated from the expression of 12 pyroptosis-related lncRNAs was evaluated by various clinical features including survival status, tumor microenvironment, clinicopathological characteristic, and chemotherapy. It may offer a significant basis for future studies.

## Introduction

Bladder cancer (BLCA) is the sixth most common cancer in men that is characterized by high risks of recurrence and mortality (1). As a common malignant tumor of the urinary tract, the 5-year overall survival rate of BLCA ranges from 23% to 48% (2, 3). Cisplatin-based chemotherapy, immunotherapy, and surgery are the most effective approaches and standard of care for BLCA (4). Novel therapeutic targets are necessary to ameliorate survival time of patients due to the limitations of BLCA treatments. Therefore, there is an urgent need for reliable new prognostic models to make targeted therapy more feasible. However, well-accepted prognostic biomarkers for BLCA are still lacking. As a result, urologists have difficulty in distinguishing the risk of BLCA patients and determine accurate treatment decisions (5).

The phenomenon of pyroptosis was first observed in the 1990s (6–9). Pyroptosis is one of the programmed cell necroses triggered and activated by some inflammasomes, which played an important role in the development of cancers. It was reported that pyroptotic death signaling was inhibited in human papillomavirus (HPV)-infected cervical cancer cells, which was correlated with poor clinical outcomes in cervical cancer (10). On the other hand, some chemotherapy drugs such as cisplatin could kill cancer cells by inducing pyroptosis (11). Pyroptosis was also found to be associated with tumor immune microenvironment. Recently, a study showed that pyroptotic cancer death could activate tumor-associated T-cell and dendritic cell (DC) infiltrations (12). It was reported that Gasdermin-D (GSDMD) was essential for effector CD8+ T cell to respond to lung cancer as well (13). These results suggested that pyroptosis was associated with the oncogenesis, immune microenvironment, and prognosis of cancer.

Long noncoding RNAs (lncRNAs) are a type of RNA more than 200 nucleotides long, which do not code for proteins (14). LncRNAs contribute to regulating the mechanisms associated with epigenetic modifications, transcriptional and posttranscriptional processes, and immune microenvironment in many diseases (15–19). Accumulated evidence has illustrated that lncRNAs are intimately related to cancer (20). For instance, lncRNA UCA1 could improve the survival of BLCA cells and reshape the tumor microenvironment (21). In addition, lncRNA ADAMTS9-AS2 enhanced cisplatin sensitivity in gastric cancer and suppressed tumor growth by mediating pyroptotic cell death (22). Moreover, lncRNA-XIST promoted non-small cell lung cancer progression by mediating pyroptotic cell death (23). Therefore, we believed that pyroptosis-related lncRNAs might participate in tumor cell proliferation and migration in cancer. However, the exact mechanism of pyroptosis-related lncRNAs in BLCA remains to be elucidated clearly.

In this study, we combined two kinds of biomarkers to construct a diagnostic model for cancers that was superior to simple genes. Few studies have confirmed the role of lncRNAs in this situation. We applied a novel modeling algorithm, paring, and iteration to construct a pyroptosis-related lncRNA signature. As well, we estimated its predictive value, diagnostic effectiveness, chemotherapeutic efficacy, immunotherapy efficacy, and tumor immune infiltration for patients with BLCA.

## Materials and Methods

### Microarray Datasets

The RNA sequencing (RNA-seq) of BLCA patients with clinical features was obtained from The Cancer Genome Atlas (TCGA) (https://portal.gdc.cancer.gov/repository). Thirty-seven pyroptosis-related genes (GPX4, NLRP7, NLRP2, CASP3, CASP6, TNF, IL1B, IL18, CASP8, NLRP6, IL6, GSDMA, GSDMC, PYCARD, CASP5, AIM2, NOD2, NLRC4, NLRP3, CASP4, CASP1, PRKACA, ELANE, TIRAP, SCAF11, PJVK, CASP9, NOD1, PLCG1, NLRP1, GSDME, GSDMD, GSDMB, P2RX7, NAIP, CLPS, and TLR4) were selected for further analysis. Co-expression pyroptosis-related lncRNAs were identified with the cutoff criteria of Pearson |R|>0.3 and P value <0.001.

### Identifying Differentially Expressed Genes and Functional Enrichment

R “limma” package was used to identify differently expressed genes (DEGs). The cutoff criterion was set as log fold change (FC) >1.5 along with false discovery rate (FDR) <0.05. Gene Ontology (GO) analysis and Kyoto Encyclopedia of Genes and Genomes (KEGG) pathway analysis were conducted by “clusterProfiler” package. As well, FDR <0.05 was considered statistically significant.

### Establishment of a Risk Model to Evaluate the Risk Score

The least absolute shrinkage and selection operator (LASSO), a machine-learning algorithm, was performed by “glmnet” package. Afterward, Cox proportional hazards regression analysis was conducted for the model. The area under the curve (AUC) value was also calculated. The highest point indicated the maximum AUC value. The procedure of calculation was terminated while the model was chosen as the optimal candidate. The 1-, 2-, 3-, 4-, and 5-year receiver operating characteristic (ROC) curves were plotted. Then, we applied a formula to define risk score:


RiskScore=ΣβiSi

We distinguished high or low risk of Risk Scores by the AUC values of the 5-year ROC curve.

### Calculation of Tumor Microenvironment Cell Infiltration

TIMER, XCELL, QUANTISEQ, MCPCOUNTER, EPIC, and CIBERSORT R script were applied to quantify the relative proportions of infiltrating immune cells (24). We used Spearman’s rank correlation analysis when exploring the relationship between the risk score values and the immune infiltrated cells.

### Prediction of Response to Chemotherapy

The R package of pRRophetic was used to predict IC50 of common chemotherapeutic agents (25). IC50 indicates the effectiveness of a substance in inhibiting specific biological or biochemical functions. The difference between groups was tested by Wilcoxon signed-rank test.

### Statistical Analysis

Correlation coefficients were computed by Spearman analyses. The Kaplan–Meier method, Cox regression model, and log-rank tests were used to evaluate the prognostic value. All statistical analyses were two-sided, and P < 0.05 was regarded as statistically significant. All statistical analyses were performed by R (version 4.0.3).

### RNA Extraction and qRT-PCR

TRIzol reagent (Invitrogen, USA) was used to isolate total RNA from tissues. HiScript II (Vazyme, China) was used to synthesize cDNA. Primers for qRT-PCR were provided by TSINGKE Biological Technology. Beta-actin was chosen as the internal reference. Expression levels of lncRNAs were calculated with 2^−ΔΔCT^. The primers used for PCR were exhibited in **Table S1**.

### Patients and Tissue Samples

The study was approved by the ethical committee of Jiangsu Cancer Hospital. Thirty-one patients with multiple BLCA who accepted partial and radical cystectomy at Jiangsu Cancer Hospital from December 2018 to August 2020 were recruited. All patients were diagnosed with urothelial carcinoma. Thirty-one tumor samples and normal bladder tissues were achieved for the experiment. Detailed clinical data were collected from the electronic medical records retrospectively. Sequencing was conducted using the Illumina PE150 platform at Novogene Bioinformatics Technology Co., Ltd.

## Results

### Identification of Co-Expressed Pyroptosis-Related Long Noncoding RNAs

**Figure 1:**
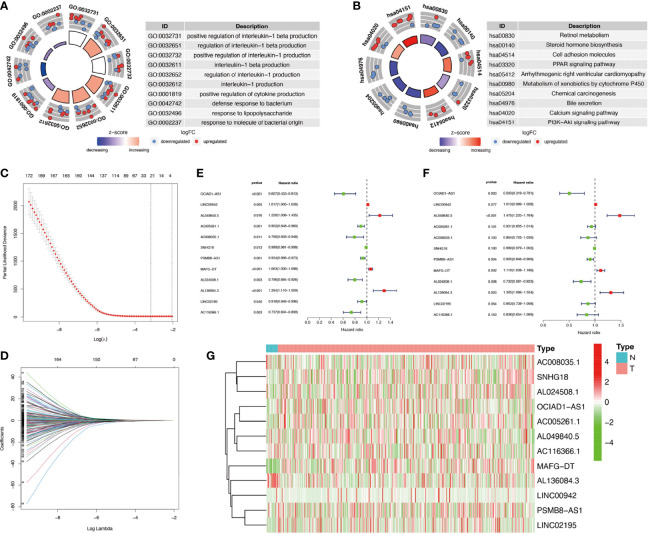
Identification of co-expressed pyroptosis-related lncRNAs. **(A, B)** Circle plots of Gene Ontology (GO) terms and Kyoto Encyclopedia of Genes and Genomes (KEGG) pathway analysis for pyroptosis-related genes. The outer circle of circle plots showed a scatter plot for each term of the logFC of the assigned genes. Red circles represented upregulation, and blue ones represented downregulation. **(C, D)** Identification of overall survival (OS)-related pyroptosis lncRNAs using the least absolute shrinkage and selection operator (LASSO) regression algorithm. **(E, F)** A forest map showed 12 pyroptosis-related lncRNAs identified by Cox proportional hazards regression in the meta-data cohort and validation cohort. **(G)** The heatmap of pyroptosis-related lncRNAs based on their expression levels.

The flowchart of this study was shown in **Figure S1**. Firstly, we obtained 37 pyroptosis-related genes from literature review. GO and KEGG analysis were carried out to investigate the function of these pyroptosis-related genes. The top 10 enriched GO and KEGG terms were shown in **Figures 1A, B**. The GO results indicated that pyroptosis-related genes were enriched in he regulation of cytokine, especially interleukin-1 (IL-1). The KEGG results indicated that pyroptosis-related genes were enriched in Peroxisome proliferator-activated receptor (PPAR) signaling pathway, calcium signaling pathway, phosphatidylinositol-3-kinase(PI3K) protein kinase B(AKT) (PI3K–AKT) signaling pathway, and so on. Then, we detected the relationship between pyroptosis-related mRNAs and pyroptosis-related lncRNAs to identify co-expressed lncRNAs. Next, the LASSO regression was conducted to identify OS-related pyroptosis lncRNAs. Twelve lncRNAs were selected for further analysis (**Figures 1C, D**). The detailed information of lncRNAs for constructing the pyroptosis-related prognostic signature was shown in **Table 1**. Univariate Cox regression analysis was conducted to analyze the prognostic values of pyroptosis-related lncRNAs in meta-data cohort and validation cohort (**Figures 1E, F**). The expressions of these 12 lncRNAs were shown in **Figure 1G** as well.

**Table 1:** The detailed information of lncRNAs for constructing the pyroptosis-related prognostic signature.

Gene	Ensemble ID	Location	β (Cox)	HR	P
OCIAD1-AS1	ENSG00000248256.1	chr4:48,852,008-48,860,203	-0.6881175	0.60692147	0.00082398
LINC00942	ENSG00000249628.3	chr12:1,500,525-1,504,424	0.01300705	1.01658149	0.00452522
AL049840.5	ENSG00000269958.1	chr14:103,696,353-103,697,163	0.38852869	1.22031272	0.01611728
AC005261.1	ENSG00000268205.1	chr19:57,304,305-57,308,562	-0.0715262	0.90203259	0.00115463
AC008035.1	ENSG00000272369.1	chr12:46,537,502-46,652,550	-0.1459938	0.79041647	0.01132062
SNHG18	ENSG00000250786.2	chr5:9,546,200-9,550,609	-0.0098145	0.98915525	0.01298739
PSMB8-AS1	ENSG00000204261.9	chr6:32,844,108-32,846,484	-0.0994647	0.93392805	0.00108726
MAFG-DT	ENSG00000265688.2	chr17:81,927,829-81,930,753	0.1041579	1.06325755	0.0001518
AL024508.1	ENSG00000272189.1	chr6:136,550,661-136,552,554	-0.3126264	0.79582983	0.00313973
AL136084.3	ENSG00000267026.5	chr9:98,847,231-98,872,403	0.26628244	1.29445181	0.0009828
LINC02195	ENSG00000236481.1	chr16:26,584,755-26,594,813	-0.1477065	0.91785548	0.03986348
AC116366.1	ENSG00000234290.2	chr5:132,468,890-132,473,043	-0.1792917	0.73672008	0.00259579

HR, hazard ratio; lncRNA, long noncoding RNA.

### Expression Level of Long Noncoding RNAs in Bladder Cancer Cell Lines

**Figure 2:**
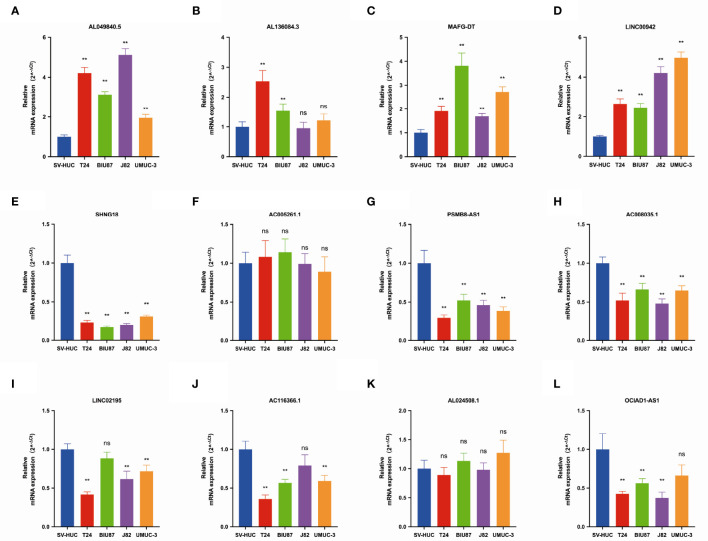
The qRT-PCR results of 12 lncRNAs in four bladder cancer cell lines. Notes: **(A)** qRT-PCR result of AL049840.5; **(B)** qRT-PCR result of AL136084.3; **(C)** qRT-PCR result of MAFG-DT; **(D)** qRT-PCR result of LINC00942; **(E)** qRT-PCR result of SNHG18; **(F)** qRT-PCR result of AC005261.1; **(G)** qRT-PCR result of PSMB8-AS1. **(H)** qRT-PCR result of AC008035.1. **(I)** qRT-PCR result of LINC02195. **(J)** qRT-PCR result of AC116366.1. **(K)** qRT-PCR result of AL024508.1. **(L)** qRT-PCR result of OCIAD1-AS1. **P < 0.01. ns, not statistically significant.

We further validated the expression of 12 pyroptosis-related lncRNAs in BLCA cells T24, BIU87, J82, and UMUC3 by qRT-PCR. As shown in **Figure 2**, we could observe that AL049840.5, AL136084.3, MAFG-DT, and LINC00942 were significantly upregulated in BLCA cell lines compared to those in SV-HUC cells. Meanwhile, the expressions of SNHG18, OCIAD1-AS1, AC008035.1, LINC02195, AC116366.1, and OCIAD1-AS1 were significantly lower in BLCA cell lines. However, no clear trend was noticed in the expression level of AL024508.1 and AC005261.1 in BLCA cell lines.

### Construction of the Risk Assessment Model

**Figure 3:**
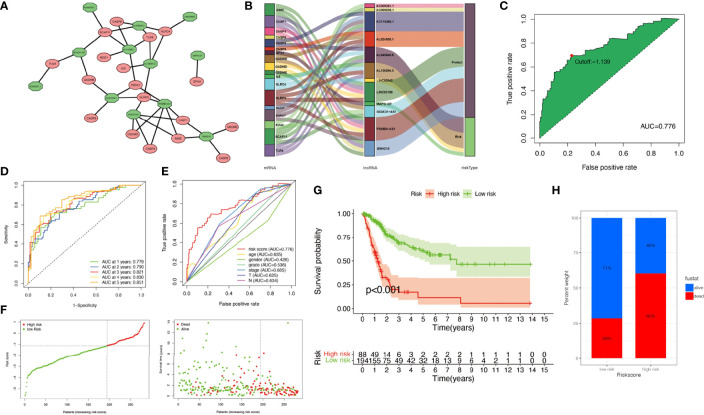
Construction of risk signature in the meta-data cohort. **(A)** The relationship between pyroptosis-related genes (red circle) and lncRNAs (green circle). **(B)** The alluvial diagram was used to visualize the attribute changes of pyroptosis-related mRNAs. **(C)** Receiver operating characteristic (ROC) curves were plotted for examining the most predictive efficacy of the signature. **(D)** Area under the curve (AUC) of time-dependent ROC curves verified the prognostic performance of the risk score. **(E)** A comparison of 5-year ROC curves with other common clinical characteristics showed the superiority of the riskScore. **(F)** Distribution of patients in The Cancer Genome Atlas (TCGA) cohort based on the median risk score and survival status for each case. **(G)** Kaplan–Meier curves of the overall survival (OS) between low- and high-risk groups. **(H)** Rate of clinical outcome in the low- and high-risk groups.

The relationship between pyroptosis-related mRNAs and 12 pyroptosis-related lncRNAs was exhibited in **Figure 3A**. The alluvial diagram was used to visualize the attribute changes of pyroptosis-related mRNAs (**Figure 3B**). In the meta-data cohort, we found that the highest point of the AUC for each ROC curve was 0.776 (**Figure 3C**). Next, we observed that AUC values of 1-, 2-, 3-, 4-, and 5-year curves were all over 0.75 (**Figure 3D**). We collected data of 403 patients with BLCA from TCGA and calculated the risk scores for all of them. Several clinical characteristics including risk score curves were also conducted (**Figure 3E**). Then, 126 cases were identified as high-risk group, and 277 cases were identified as low-risk group based on the cutoff point confirmed previously. Risk scores and survival status of each patient were depicted in **Figure 3F**. Obviously, we could observe that patients in the low-risk group showed a significant survival advantage that was consistent with the result of Kaplan–Meier analysis (**Figures 3G, H**). Similar results were obtained in the validation cohort and the whole cohort (**Figure S2**).

### Relationship Between Clinical Characteristics and Risk Score

Clinical characteristics of the two groups were also exhibited in **Figure S3**. Clinicopathological characteristics of patients in TCGA-BLCA cohort were displayed in **Table 2**. We observed that most clinical features were significantly related to the risk score. As well, we found that age [P < 0.001, hazard ratio (HR) = 1.038, 95% CI 1.019–1.056], clinical stage (P < 0.001, HR = 1.941, 95% CI 1.543–2.441), T stage (P < 0.001, HR = 1.744, 95% CI 1.365–2.227), and risk score (P < 0.001, HR = 1.941, 95% CI 1.682–2.240) showed statistical differences by univariate Cox regression analysis in the meta-data cohort and validation cohort (**Figures S4A, B**). Therefore, based on the C-index values, a nomogram integrating the risk score, age, gender, clinical stage, tumor differentiation, and TNM stage was constructed (**Figure S4C**). The nomogram-predicted results were consistent with the survival status of the patients (**Figures S4D–F**). The result of decision curve analysis (DCA) further elucidated that the risk score served as the most exact prognostic indicator among clinical variables in clinical decision-making (**Figure S4G**).

**Table 2:** Clinicopathological characteristics of patients in TCGA-BLCA cohort.

Characteristic	TCGA-BLCA cohort (n = 407)	
	Number	%
**Age (years)**		
≤65	161	39.6
>65	246	60.4
**Gender**		
Male	301	74.0
Female	106	26.0
**Histological grade**		
Low stage	20	5.0
High stage	384	94.3
Unknow	3	0.7
**Pathological stage**		
Stage I	2	0.5
Stage II	129	31.7
Stage III	141	34.6
Stage IV	133	32.7
Unknown	2	0.5
**T stage**		
T1	4	1.0
T2	118	29.0
T3	194	47.7
T4	58	14.3
Unknown	33	8.0
**N stage**		
N0	237	58.2
N1	45	11.1
N2	76	18.6
N3	7	1.7
NX	42	10.4
**M stage**		
M0	195	47.9
M1	11	2.7
MX	201	49.4
**Outcome**		
Alive	250	61.4
Dead	157	38.6

BLCA, bladder cancer; TCGA, The Cancer Genome Atlas.

**Figure 4:**
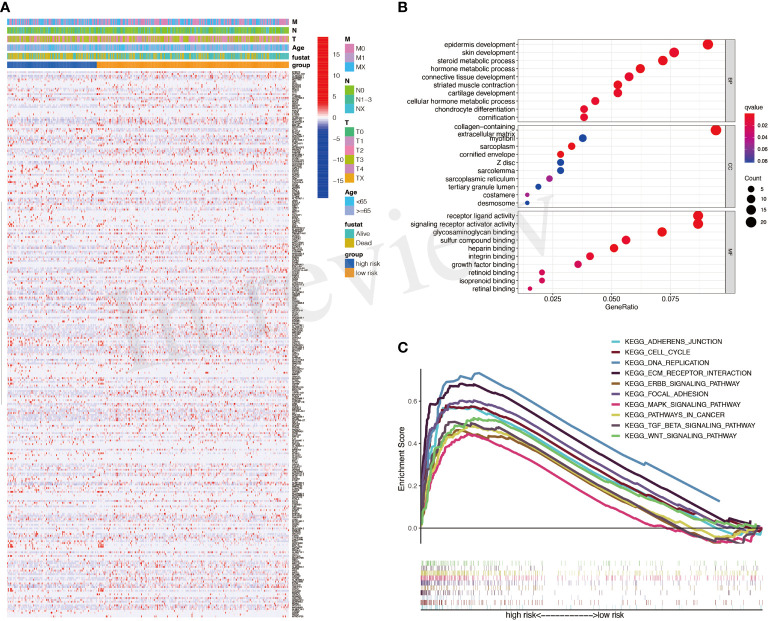
Differentially expressed genes (DEGs) between high- and low-risk groups. **(A)** A heatmap for DEGs between high- and low-risk groups. **(B)** Gene Ontology (GO) analysis for DEGs. **(C)** Activated pathways analyzed by gene set enrichment analysis (GSEA).

DEGs between high- and low-risk groups were identified and exhibited in **Figure 4A**. The GO results showed that DEGs were enriched in many metabolic processes, such as steroid and hormone (**Figure 4B**). The gene set enrichment analysis (GSEA) results showed that mitogen-activated protein kinase (MAPK) signaling, transforming growth factor (TGF)-β signaling, and WNT signaling were with important significance in the high-risk group (**Figure 4C**).

### Estimation of Tumor Microenvironment and Chemotherapeutics

**Figure 5:**
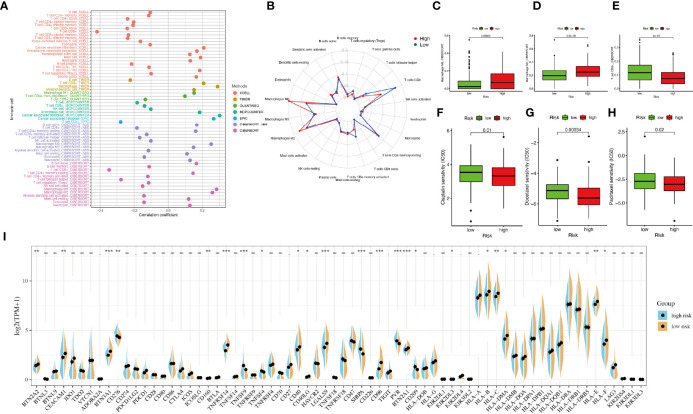
Tumor-infiltrating immune cells and chemotherapeutics. **(A)** The relationship between immune cells and riskScore. Each color represented a distinct algorithm. **(B)** The proportions of immune cells in the high- and low-risk groups. **(C–E)** The proportions of macrophages M0, macrophages M2, and CD8+ T cell in the high- and low-risk groups. **(F–H)** The half-maximal inhibitory concentration (IC50) of 3 common chemotherapeutic agents (cisplatin, docetaxel, and paclitaxel). **(I)** The expression of 68 immune checkpoint genes in the high- and low-risk groups. *P < 0.05, **P < 0.01, ***P < 0.001.

The correlation between immune cells and riskScore was calculated as well (**Figure 5A**). The immune infiltration results of the two groups were shown in **Figure 5B**. The proportions of M0 macrophages and M2 macrophages in the high-riskscore group were significantly higher than those in the low-riskscore group. However, the proportion of CD8+ T cells was significantly lower in the high-riskscore group (**Figures 5C–E**).

Based on the pRRophetic algorithm, we explored the relationship between riskscore and chemoresistance by calculating the half-maximal inhibitory concentration (IC50) of 3 common chemotherapeutic agents (cisplatin, docetaxel, and paclitaxel) for BLCA. We observed that patients in the high-riskscore group were more sensitive to these three chemotherapeutic agents (**Figures 5F–H**).

### Immunotherapy Analysis

**Figure 6:**
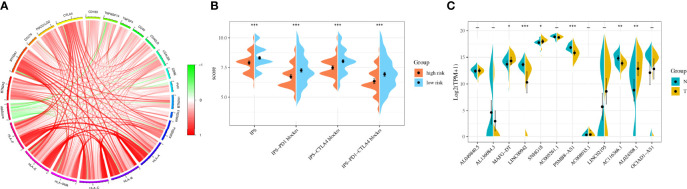
Immunotherapy analysis and validation in the original cohort. **(A)** The correlation between immune checkpoint genes and riskscore. **(B)** The IPS, IPS-PD1 blocker, IPS–CTLA4 blocker, and IPS–PD1–CTLA4 blocker values in the high- and low-risk groups. **(C)** The RNA sequencing (RNA-Seq) results of 12 lncRNAs in bladder cancer tissues and normal tissues. *P < 0.05, **P < 0.01, ***P < 0.001.

Considering the clinical application and benefits of immune checkpoint inhibitors, we identified 68 immune checkpoint genes from a review of the literature (26, 27). We found that the expression of many immune checkpoints showed significant differences between the two groups (**Figure 5I**). As well, riskscore was observed to be negatively correlated with BTN2A2, BTN3A1, CD276, PDCD1LG2, CTLA4, CD160, TNFRSF14, CD40, CD40LG, LGALS9, CD96, BTN2A1, KIR2DL3, KIR2DL4, HLA-A, HLA-B, HLA-C, HLA-DMA, HLA-E, and HLA-F and positively correlated with TNFSF4 and PVR (**Figure 6A**). As described in **Figure 6B**, the scores of IPS, IPS−PD1 blocker, IPS−CTLA4 blocker, and IPS−PD1 −CTLA4 blocker were lower in the high-risk group. All these results suggested that risk score may be related to immunotherapy.

### Validation of Long Noncoding RNAs and Riskscore in the Original Cohort

We used 31 primary BLCA samples and 31 normal bladder tissues for microarray analysis. The clinical characteristics were described in **Table 3**. The results showed that the expressions of MAFG-DT, SNHG18, and AL024508.1 were higher in tumor tissues, while the expressions of LINC00942, PSMB8-AS1, and AC116366.1 were higher in normal tissues (**Figure 6C**). Riskscores were also calculated by the same formula. Clinical characteristics of the two groups were also exhibited in **Figure S5**.

**Table 3:** Clinicopathological characteristics of patients in the original cohort.

Characteristics	Case	riskscore		P value
		Low	High	
**All cases**	31	15	16	
**Age**				1
< 65	13	6	7	
≥ 65	18	9	9	
**Gender**				1
Male	24	12	12	
Female	7	3	4	
**T stage**				0.034
Ta	4	4	0	
T1	9	2	7	
T2	9	5	4	
T3	6	4	2	
T4	3	0	3	
**N stage**				0.484
N0	27	13	14	
N1	0	0	0	
N2	2	0	2	
N3	1	1	0	
Nx	1	1	0	
**M stage**				0.461
M0	26	12	14	
M1	4	3	1	
Mx	1	0	1	
**Grade**				0.654
Low	6	2	4	
High	25	13	12	
**Smoke**				0.473
Yes	12	7	5	
No	19	8	11	
**Recurrence**				
Yes	10	5	5	1
No	21	10	11	

## Discussion

As far as we are aware, our research is the first comprehensive and detailed analysis of pyroptosis-related lncRNAs in BLCA that could contribute to offering a significant basis for future studies.

To begin with, we retrieved raw data of lncRNAs and transcripts from TCGA. The functional analyses indicated that the pyroptosis-related genes were associated with the regulation of cytokine, especially IL-1. Caspase-1, which could initiate pyroptosis, could mediate the maturation and secretion of IL-1 as well (28). The activation of pyroptosis led to the release of the inflammatory mediator IL-1, which promoted the occurrence of cancer in many ways (29). As well, the functional analyses indicated that many pathways such as PPAR signaling pathway, calcium signaling pathway, and PI3K–AKT signaling pathway contributed to the process. It was confirmed that Cyclin A2 and cyclin-dependent kinase dysregulated GSDMD through the inhibition of PI3K–AKT pathway in gastric cancer (30).

Then, we compared the mRNA expression levels of 37 well-accepted pyroptosis-related genes between BLCA and normal tissues. Interestingly, we found that most of these genes were expressed aberrantly, which suggested that pyroptosis contributed to the development of BLCA. Considering the role of lncRNAs, we identified 172 co-expressed pyroptosis-related lncRNAs.

Afterward, we constructed a 12-lncRNA risk signature to explore the relationship between BLCA and pyroptosis-related lncRNAs. Among these selected lncRNAs, updates confirm that some of them may play different functional roles in the progress of cancer. For instance, lncRNAs OCIAD1-AS1, LINC02195, MAFG-DT, AL136084.3, and PSMB8-AS1 were with great prognostic value for BLCA (31–34). As discovered by Sun et al. (35), lncRNA LINC00942 exerted its functions as an oncogene in promoting METTL14-mediated m6A methylation and regulating the expression and stability of its target genes CXCR4 and CYP1B1 in BRCA initiation and progression. Fan et al. (36) found that lncRNA SNHG18 facilitated non-small cell lung cancer growth and metastasis by modulating the miR-211-5p/BRD4 and may be a potential therapeutic target for the treatment.

Not only did we count each AUC value of ROC to get the most accurate model, but also getting the optimal cutoff point distinguished the high- or low-risk group among patients with BLCA. Obviously, patients in the high-risk group showed a significant survival disadvantage by using Kaplan–Meier analysis. Surprisingly, we found that riskscore was related to gender and age. Moreover, patients with high-grade BLCA had significantly higher riskscores than those with low-grade BLCA. The riskscore of patients with different T, N, M, and clinical stage also showed a significant difference that was consistent with prognosis. Therefore, a nomogram integrating these characteristics was constructed that could predict clinical outcomes well.

After identifying differently expressed genes between high- and low-risk groups, GSEA results also showed that the pathways of adherens junction, cell cycle, DNA replication, extracellular matrix (ECM) receptor signaling, epidermal growth factor receptor (ERBB) signaling, focal adhesion, MAPK signaling, TGF-β signaling, and WNT signaling were enriched in the high-risk group. This suggests that pyroptosis-related lncRNAs in BLCA may be related to pathways such as MAPK signaling, TGF-β signaling, and WNT signaling. For example, miR-186-5p suppressed BLCA cell proliferation, migration, invasion, and epithelial-mesenchymal transition (EMT) process by targeting RAB27A/B to inactivate the MAPK signaling (37). Moreover, grape seed proanthocyanidins effectively inhibited the migration and invasion of bladder cancer (BC) cells by reversing EMT through suppression of the TGF-β signaling pathway (38). As Gao and Ji (39) suggested, LINC00707 contributed to the proliferation and metastasis of BLCA by activating Wnt/β-catenin signaling. Pathways such as ECM receptor signaling, ERBB signaling, and focal adhesion were associated with the tumor microenvironment.

The results of immune infiltration analysis indicated that the risk score exhibited a positive correlation with the infiltration of B cells naive, CD4+ T cells, DCs, macrophages M0, and macrophages M2. In a previous study, CD4+ T cells were proven to be more recruited by BLCA cells, which promoted the BLCA metastasis (40). Both DCs and CD4+ T cells were proven related to Bacillus Calmette Guerin (BCG)-induced immune response, which indicated the importance of tumor-infiltrating immune cells in BLCA progression and therapy (41).

Response to immune checkpoint inhibitors [such as cytotoxic T lymphocyte-associated antigen-4 (CTLA4)] is the key to satisfying treatment. However, not every patient could benefit from immunotherapy (42). In our study, we surprisingly observed that the expressions of many immune checkpoint genes, including CTLA4, showed significant differences between the two groups. The riskscore model was negatively correlated with most immune checkpoint genes. As well, the scores of IPS, IPS−PD1 blocker, IPS−CTLA4 blocker, and IPS−PD1−CTLA4 blocker were lower in the high-risk group. It has been reported that IPS was a superior predictor of response to CTLA-4 and anti-PD-1 (43). All results above suggested that our model may be a potential index for evaluating the response to immunotherapy in patients with BLCA. Besides, we also detected the response to chemotherapy sensitivity of patients by calculating IC50 value and screen out candidate small-molecule compounds. Collectively, these discoveries may offer suitable treatment alternatives for BLCA patients.

Overall, our research indicated that pyroptosis is closely connected to BLCA. Moreover, the score based on risk signature generated from 12 pyroptosis-related lncRNAs evaluated this original model *via* various clinical settings such as TNM stage, clinicopathological characteristics, tumor microenvironment, and chemotherapy. It may offer a significant basis for future studies.

## Data Availability Statement

Publicly available datasets were analyzed in this study. These data can be found here: https://portal.gdc.cancer.gov/repository.

## Ethics Statement

The studies involving human participants were reviewed and approved by the ethical committee of Jiangsu Cancer Hospital. The patients/participants provided their written informed consent to participate in this study.

## Author Contributions

HL designed the article and wrote the article. JW and LL conducted the experiments and revised the article. XW obtained the samples and clinical information. HC contributed to the concept and revised the article. All authors contributed to the article and approved the submitted version.

## Conflict of Interest

The authors declare that the research was conducted in the absence of any commercial or financial relationships that could be construed as a potential conflict of interest.

## Publisher’s Note

All claims expressed in this article are solely those of the authors and do not necessarily represent those of their affiliated organizations, or those of the publisher, the editors and the reviewers. Any product that may be evaluated in this article, or claim that may be made by its manufacturer, is not guaranteed or endorsed by the publisher.
