# Supplementary figures and images for: Identifying a Novel Defined Pyroptosis-Associated Long Noncoding RNA Signature Contributes to Predicting Prognosis and Tumor Microenvironment of Bladder Cancer
Source: Front Immunol. 2022 Jan 27;13:803355. doi: 10.3389/fimmu.2022.803355 (PMC8828980; doi:10.3389/fimmu.2022.803355)

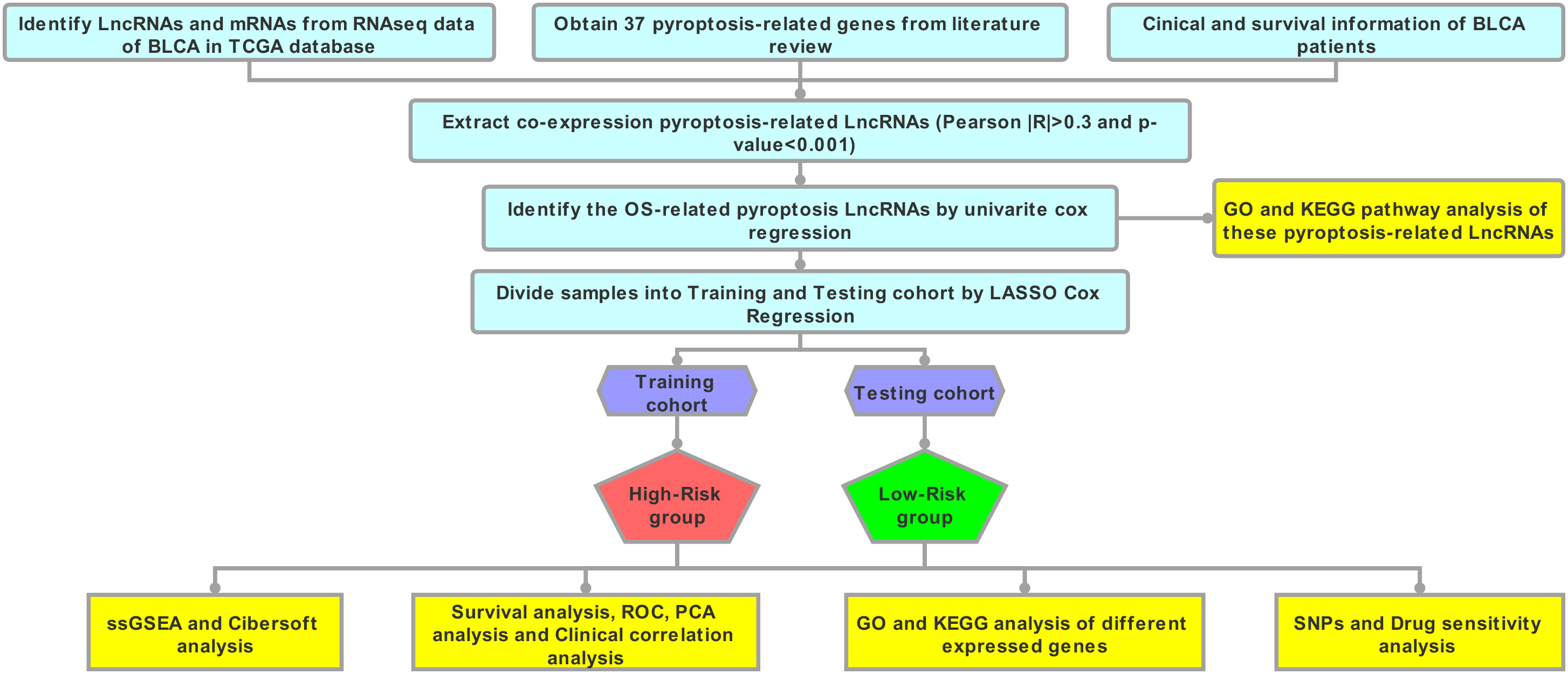

Supplement: Supplementary file 1 [file Image_1.tif]

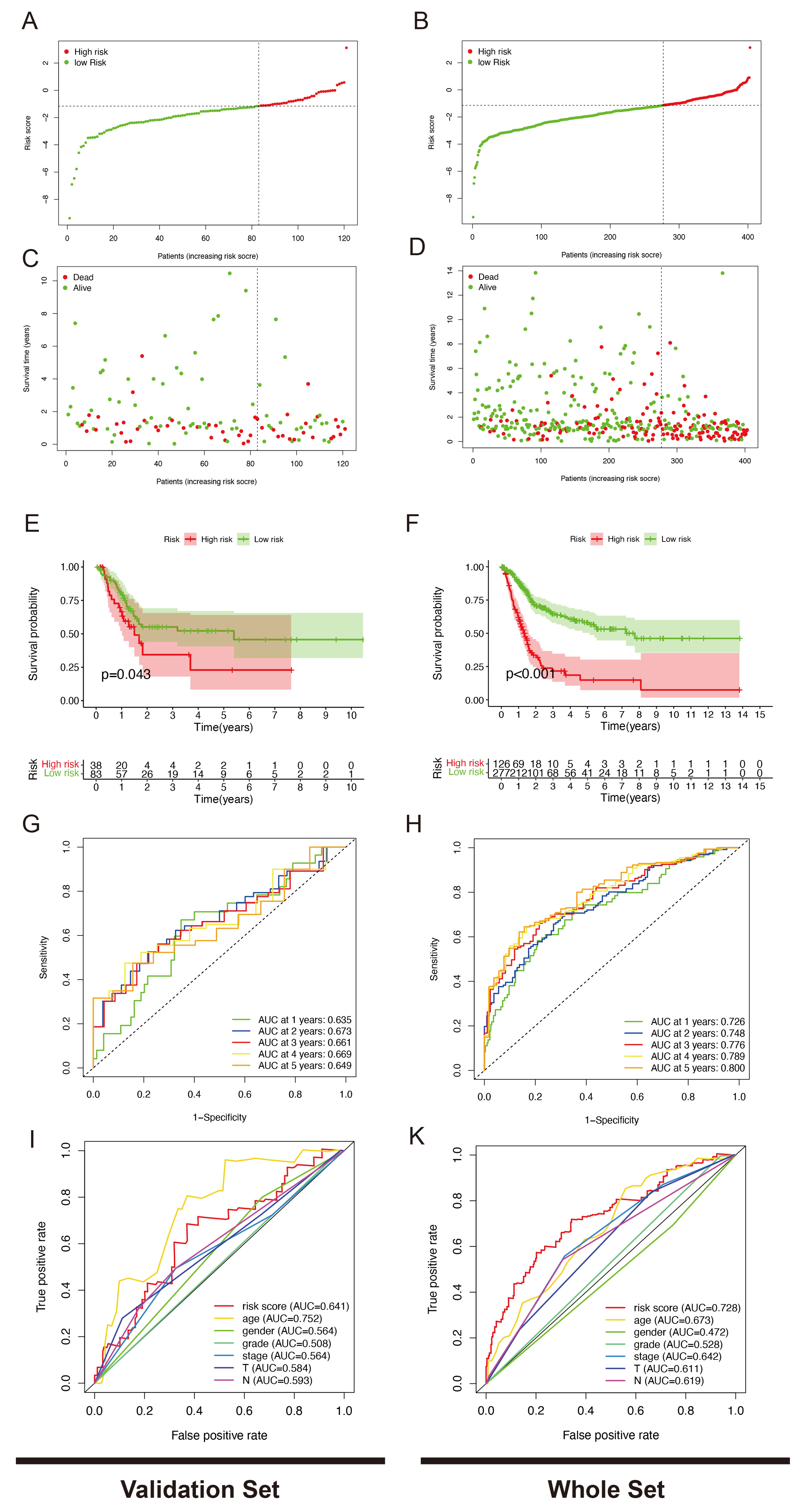

Supplement: Supplementary file 2 [file Image_2.tif]

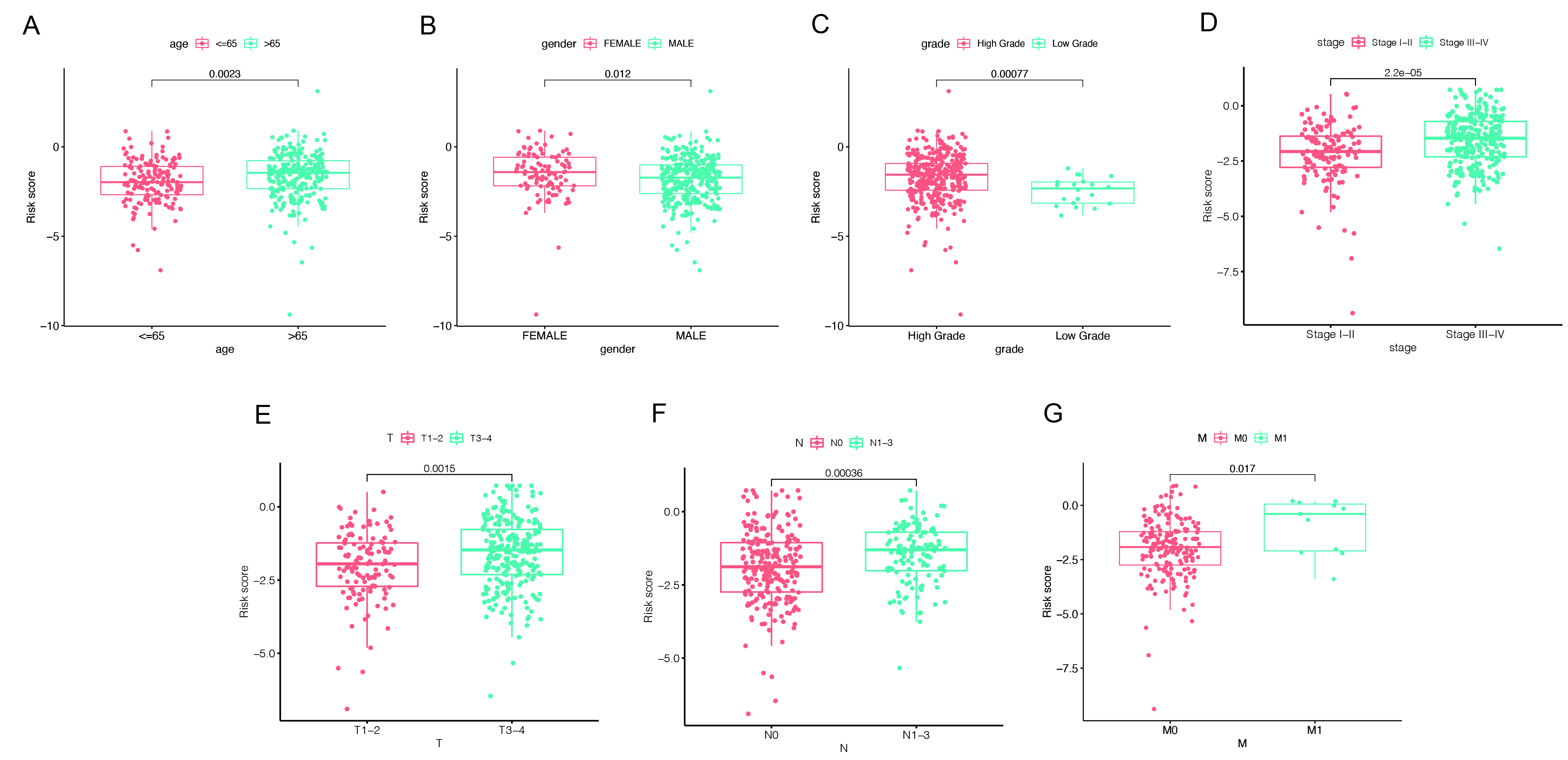

Supplement: Supplementary file 3 [file Image_3.jpeg]

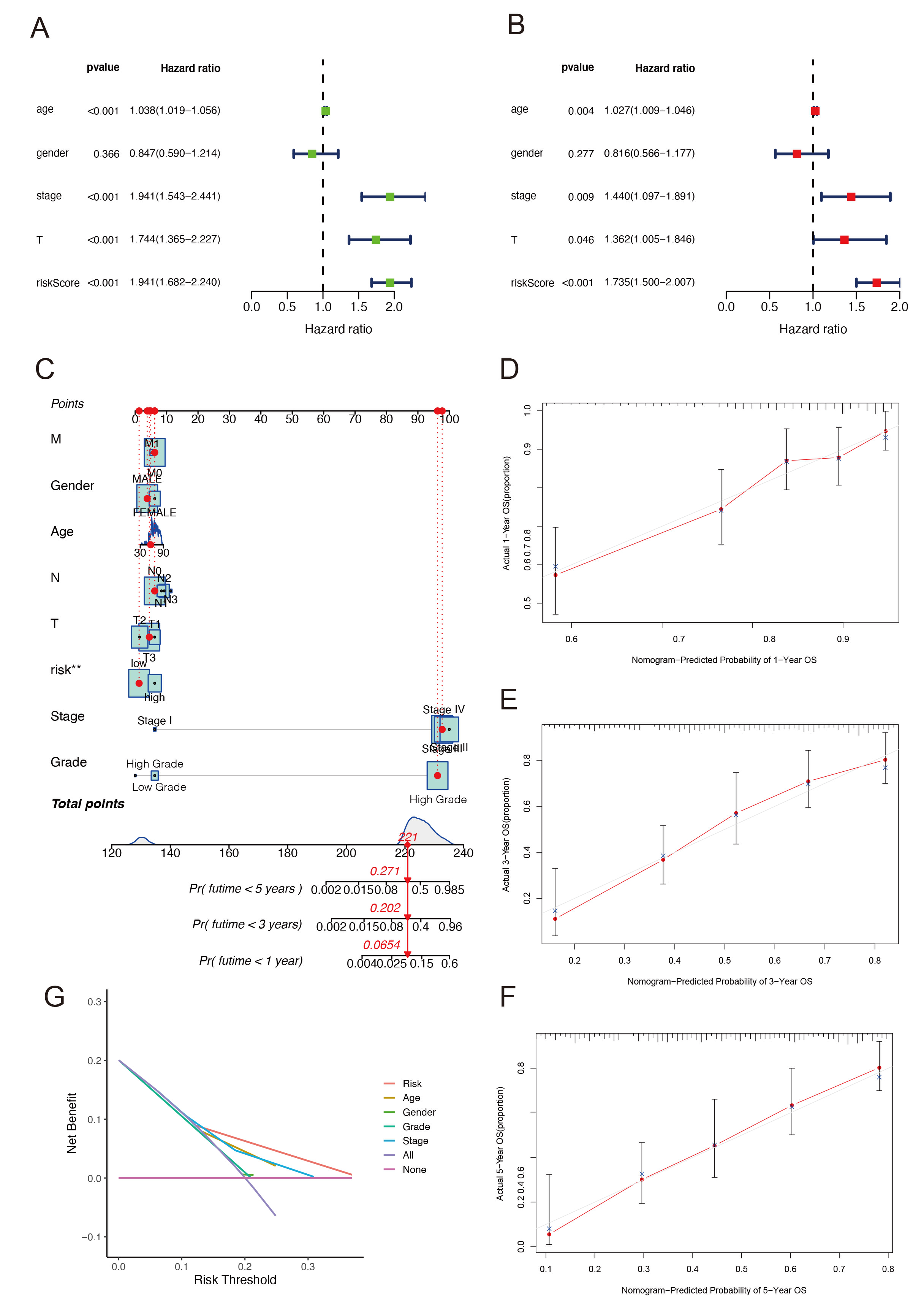

Supplement: Supplementary file 4 [file Image_4.tif]

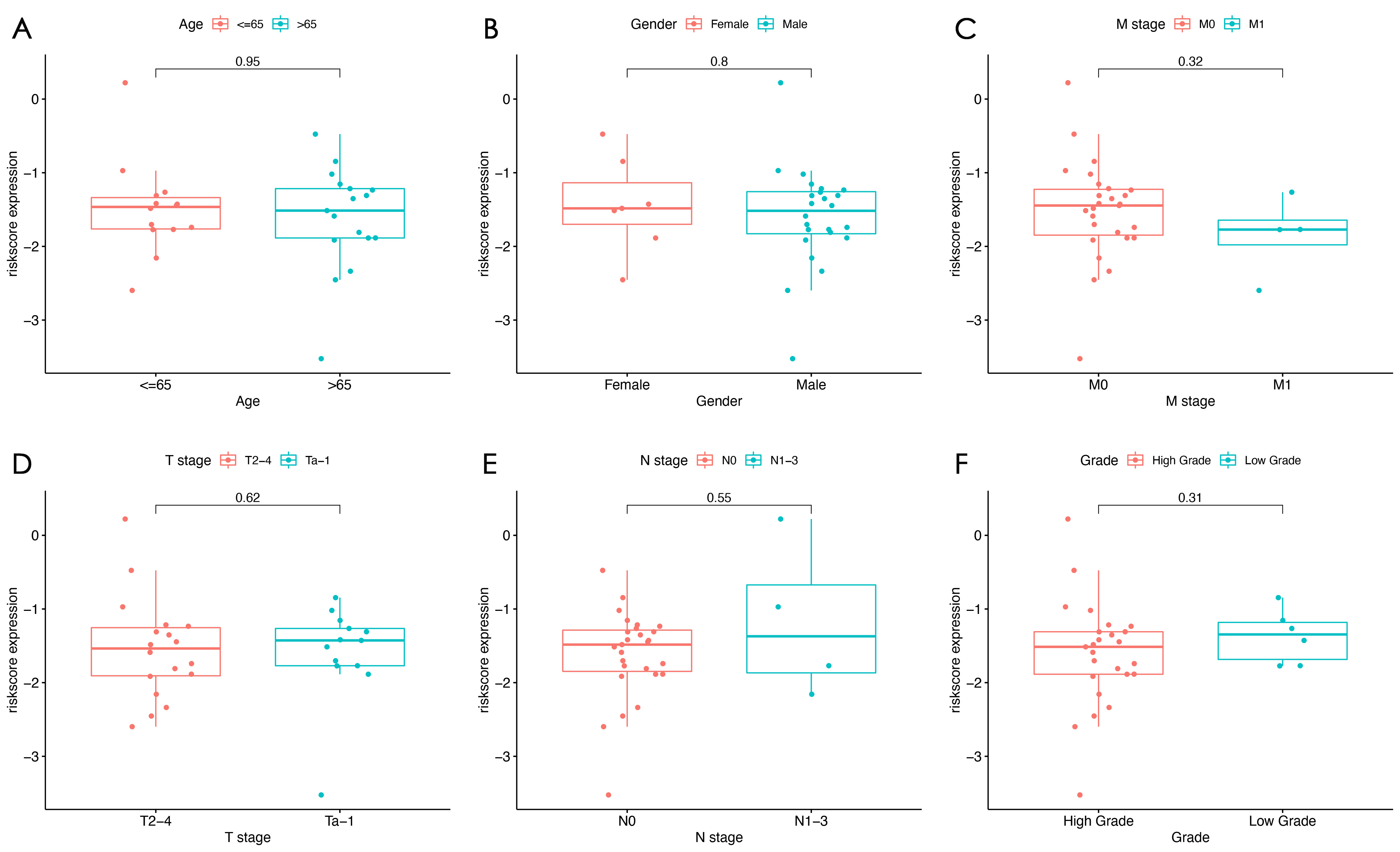

Supplement: Supplementary file 5 [file Image_5.tif]
