# Supplementary material for: Identifying a Novel Defined Pyroptosis-Associated Long Noncoding RNA Signature Contributes to Predicting Prognosis and Tumor Microenvironment of Bladder Cancer
Source: Front Immunol. 2022 Jan 27;13:803355. doi: 10.3389/fimmu.2022.803355 (PMC8828980; doi:10.3389/fimmu.2022.803355)
Supplement: Supplementary file 6 [file Table_1.docx]

**Table S1 Oligonucleotide sequences used in the present study**

| Primers | Sequences | |
| --- | --- | --- |
| AL049840.5 | Forward | CACTGACTTGTGAGCTGTG |
|  | Reverse | GTAAGTGCTCTACAGCATTCAG |
| AL136084.3 | Forward | CTATGATAAAGGATTAGGCAGTGG |
|  | Reverse | CTGGTGTTGAAAGAAGGAGG |
| MAFG-DT | Forward | GACTTCTCTGCACCTCCAG |
|  | Reverse | CTCAGTCCCACAGAAGGTG |
| LINC00942 | Forward | GTTTCCCTGGAAACACCAC |
|  | Reverse | TTGAACATGAAGGCAGGTG |
| SNHG18 | Forward | AGTGCTTGAATTTCAGCCAC |
|  | Reverse | GTCATGGATCACATCTTAAAGACC |
| AC005261.1 | Forward | CCTTTACCCTTCTCTTGAACAC |
|  | Reverse | GCTATACTCCAATACCAACACTC |
| PSMB8-AS1 | Forward | CTACTGTTCTGAGGTTTCCC |
|  | Reverse | ATAATGCAGACTTCCACCTC |
| AC008035.1 | Forward | CCTGTTACCTCATAAAGGTGC |
|  | Reverse | GAGCAGGCAATAATCTCTATTCTC |
| LINC02195 | Forward | GCTATCATATACTGGTGAAGACTC |
|  | Reverse | GAATCCTCCTACTTTGGCAG |
| AC116366.1 | Forward | TGGGACTACAGCTCTTTGC |
|  | Reverse | ACCTCAAGGCATGAGTAAGG |
| AL024508.1 | Forward | CAGTTCCGCATGGTTACAC |
|  | Reverse | TATCTTCAGCCAAGCCAGAG |
| OCIAD1-AS1 | Forward | CAGGGACAAGAACCAAGTG |
|  | Reverse | TAGGTGTCAAGGACTCTGC |
| β-actin | Forward | ATGACTTAGTTGCGTTACACC |
|  | Reverse | GACTTCCTGTAACAACGCATC |
